# Bone quality and quantity of the mandibular symphyseal region in autogenous bone grafting using cone-beam computed tomography: a cross-sectional study

**DOI:** 10.1186/s13005-021-00282-2

**Published:** 2021-07-12

**Authors:** Yaser Safi, Reza Amid, Mahdi Kadkhodazadeh, Hamed Mortazavi, Mohamad Payam Sharifi, Shiva Gandomi

**Affiliations:** 1 Department of Oral and Maxillofacial Radiology, School of Dentistry, Shahid Beheshti University of Medical Sciences, Tehran, Iran; 2 Department of Periodontics, School of Dentistry, Shahid Beheshti University of Medical Sciences, Tehran, Iran; 3 Department of Oral Medicine, School of Dentistry, Shahid Beheshti University of Medical Sciences, Tehran, Iran; 4 School of Dentistry, Shahid Beheshti University of Medical Sciences, Tehran, Iran

**Keywords:** Anatomy, Bone graft, Cone-beam computed tomography, Dental implant, Mandible

## Abstract

**Background:**

Bone volume plays a pivotal role in the success of dental implant treatment. Autogenous bone grafts should be harvested from reliable sites in the maxillofacial region. This study sought to assess the quantity and quality of bone in the mandibular symphysis for autogenous bone graft harvesting using cone-beam computed tomography (CBCT).

**Methods:**

This cross-sectional study evaluated the CBCT scans of 78 adults presenting to three oral and maxillofacial radiology centers. The vertical (VD) and horizontal (HD) alveolar bone dimensions, cortical thickness (CT), and cancellous to cortical bone ratio (C/C) were measured in the interforaminal region of the mandible at the sites of central incisor to first premolar teeth. The interforaminal distance (ID) and the anterior loop length were also measured. Nonparametric statistical tests were used to analyze the data with respect to sex, age, and tooth position.

**Results:**

The median VD, HD, and CT of the symphysis were 20.21 (3.26), 4.13 (0.37), and 2.25 (0.23) mm, respectively. The median C/C was 1.51 (0.11). The median ID was 52.24 (8.24) mm, and the median anterior loop length was 1.82 (1.06) mm. Significant differences were observed in all parameters among different teeth. Most of the measured parameters were greater in males compared with females. There were significant differences in ID, VD, and CT between different age groups.

**Conclusions:**

The quantity and quality of the available bone in the mandibular symphysis for bone graft harvesting vary by gender, age, and harvesting site, necessitating careful preoperative evaluation.

## Background

Due to the increasing use of dental implants, and the importance of alveolar bone support, reliable sites are critical for autogenous bone graft harvesting. Conditions such as long-term edentulism, aging, trauma, and systemic diseases can cause or aggravate alveolar bone loss [1]. Bone grafting is a surgical procedure performed to eliminate the dead space, minimize the risk of postoperative infection, preserve the regional contour, and improve the soft tissue and bone healing. Autogenous bone is still the gold standard for bone grafting [2].

Intramembranous autogenous bone grafts have several advantages over other grafts used in the maxillofacial region, including minimal bone loss, high-volume preservation, minimal antigenicity, and having a higher concentration of bone morphogenetic proteins [3]. Autogenous bone grafts may be harvested from the intra- or extraoral sites. The intraoral donor sites can often provide adequate bone volume for harvesting. Intraoral bone grafts are often associated with more favorable outcomes compared with extraoral bone grafts [4]. Different intraoral sites are available for bone harvesting, including the mandibular symphysis, ramus, internal and external oblique ridges, and the maxillary tuberosity [5]. The mandibular ramus is the first option for bone harvesting due to fewer postoperative complications. On the other hand, the mandibular symphysis is more easily accessible in comparison with the ramus, which is particularly important in patients with mouth opening limitation or temporomandibular disorders. Moreover, the mandibular symphysis has more cancellous bone than does the ramus [6]. Despite the benefits of the mandibular symphysis for bone harvesting, the amount of the available bone for harvesting and the vital anatomical structures in this area should be accurately determined preoperatively due to the possibility of complications such as intraoperative bleeding, mental nerve injury, and pulp necrosis of the mandibular anterior teeth.

The interforaminal distance (ID) determines the width of the harvestable bone block. Since the mandibular canal, containing the inferior alveolar nerve and the vasculature, terminates at the mental foramen, injury to this region can cause sensory disturbances in the mandibular anterior teeth and the soft tissue [7]. The height of the harvestable bone block is determined by the bone height below the apex of the mandibular anterior teeth, and the labial bone thickness determines the bone block thickness [8].

Several studies have confirmed the optimal efficacy of cone-beam computed tomography (CBCT) as a reliable tool for accurate measurement of the volume, dimensions, and quality of the donor site bone, and determination of the relative position of anatomical structures [9, 10].

Considering the existing anatomical variations in different populations, the significance of bone quality and quantity, and the existing differences in this regard among different donor sites, this study sought to assess the bone quantity in the mandibular symphysis and assess its density by evaluating the cancellous to cortical bone ratio (C/C) for autogenous bone graft harvesting in an Iranian population using CBCT.

## Methods

This was a retrospective cross-sectional study. The study protocol was approved by the institutional review board of Shahid Beheshti University of Medical Sciences (code no: IR.SBMU.DRC.REC.1397.061), and it was conducted in accordance with the Declaration of Helsinki and its subsequent revisions. The study was conducted in accordance with the STROBE statement.

### Subjects

This study was conducted on CBCT scans of adults retrieved from the archives of three oral and maxillofacial radiology centers. The CBCT scans had been requested for purposes not related to this study from 2018 to 2019. The CBCT scans had been obtained by the New Tom VGI CBCT scanner (Quantitative radiology, Verona, Italy) in two centers with the exposure settings of 110 kVp and 3.3–20 mA. The Scanora 3D CBCT scanner (Soredex, Helsinki, Finland) was used in the third center with the exposure settings of 90 kVp and 4 mA. The size of field of view was determined according to the patients’ size and referral reason.

The available optimal-quality CBCT scans (in DICOM format) of Iranian adults between 20 and 70 years with no missing anterior teeth were included in this study. Edentulous patients and those with osteoporosis or other diseases affecting the bone density, intraoral exostoses, and pathologies such as mandibular cysts or tumors were excluded from the study. Patients receiving medications affecting the bone metabolism were also excluded.

### Sample size

According to the results of a previous study [11, 12], the sample size was calculated to be 78 CBCT scans, using the following formula (α = 0.05, β = 0.2, d = 0.65,${\sigma }_{2}$=4)

$$n=\frac{{\left({Z}_{1-\alpha /2}+{Z}_{1-\beta }\right)}^{2}{\sigma }_{2}}{{d}^{2}}$$

Due to the retrospective nature of the study, the selection bias was an issue. To minimize the effect of selection bias, 600 CBCT scans were assessed for eligibility; out of which, 78 were selected according to the eligibility criteria.

### Measurements

CBCT scans were evaluated by a calibrated oral and maxillofacial radiologist with 20 years of clinical experience. Subjectivity of measurement was another source of bias in this study. To verify the reliability of measurements, the intra-observer agreement was calculated. for this purpose, all 78 CBCT scans were evaluated twice with a 3-month interval.

The primary outcome of this study was to assess the bone quality and quantity of different parts of the symphysis for bone harvesting. For this reason, the following parameters were separately measured for eight mandibular teeth (central incisor to first premolar at both sides), using OnDemand3D Project Viewer software version 10.1 with a ruler with 0.1 mm accuracy. There were no restrictions with respect to the use of image enhancement filters. The variables were all measured in millimeters.

**Fig. 1:**
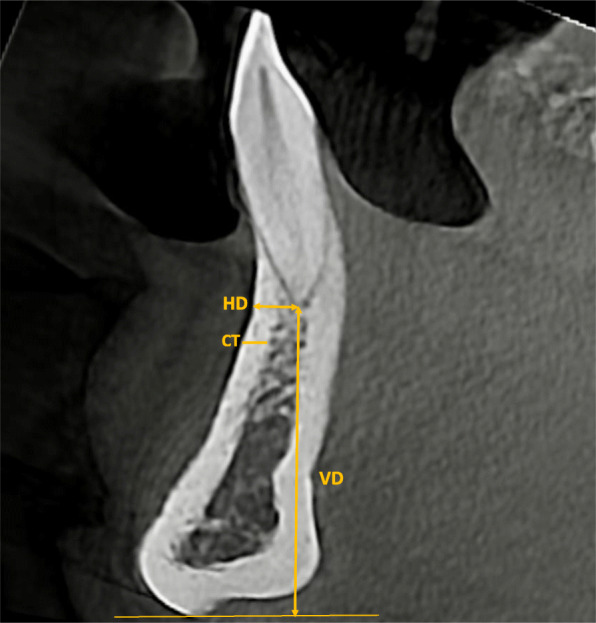
Measurement of horizontal (HD) and vertical (VD) bone dimensions and cortical bone thickness (CT) of the mandibular symphysis on a multiplanar section of the mandibular central incisor region

**Fig. 2:**
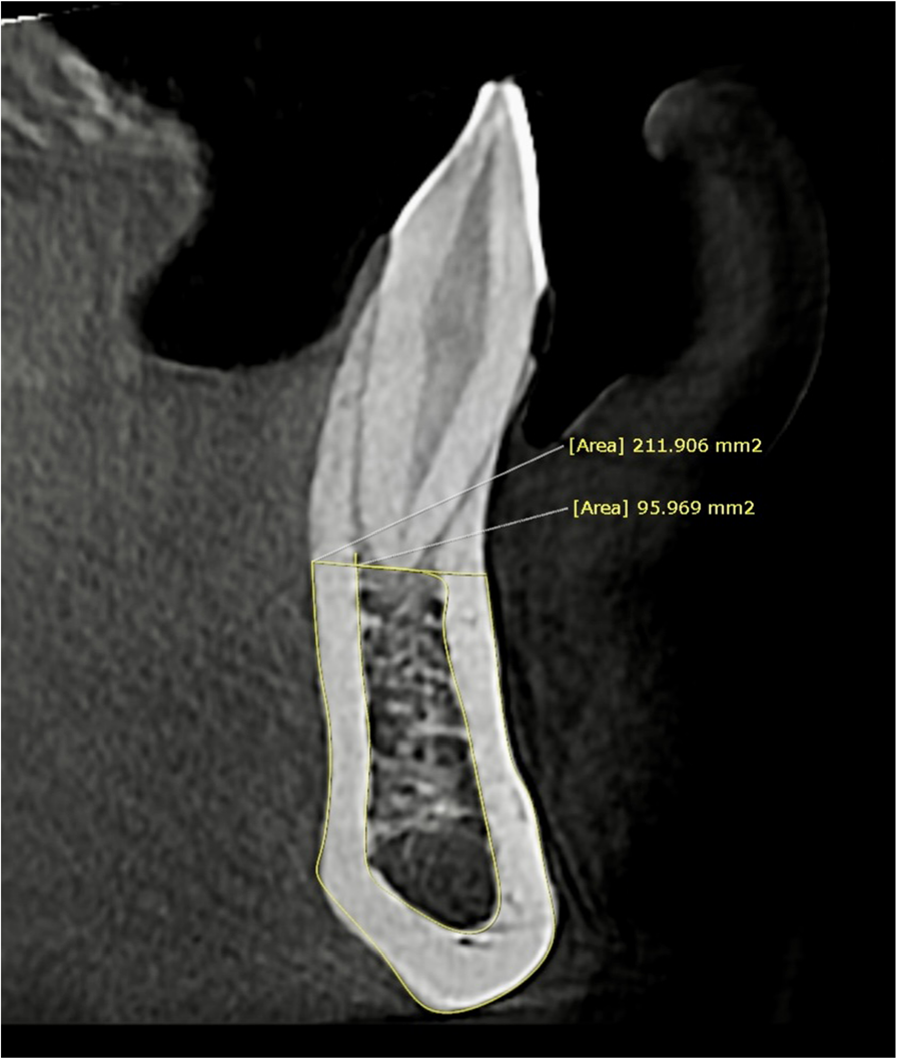
Two-dimensional calculation of cancellous to cortical bone ratio on a multiplanar section of the mandibular canine region

1. Vertical dimension of the symphysis (VD): vertical distance between the most inferior part of the border of mandible in the anterior region and the apex of the anterior teeth in the sagittal plane.
2. Horizontal dimension of the symphysis (HD): horizontal distance between the labial cortex and the apex of the anterior teeth in the sagittal plane.
3. Cortical bone thickness at the symphysis (CT): distance from the outermost buccal border of the labial plate in the anterior mandible to the outermost border of the lingual plate in the sagittal plane, 5 mm caudally from the apex and perpendicular to the plane (Fig. 1).

4. C/C in different sections of the symphysis: ratio of cancellous bone area to cortical bone area in the sagittal plane in two-dimensional calculation. For bone curvature adaptation, multiple points with a maximum distance of 1 mm were used. More points were used in areas with greater curvature (Fig. 2).

**Fig. 3:**
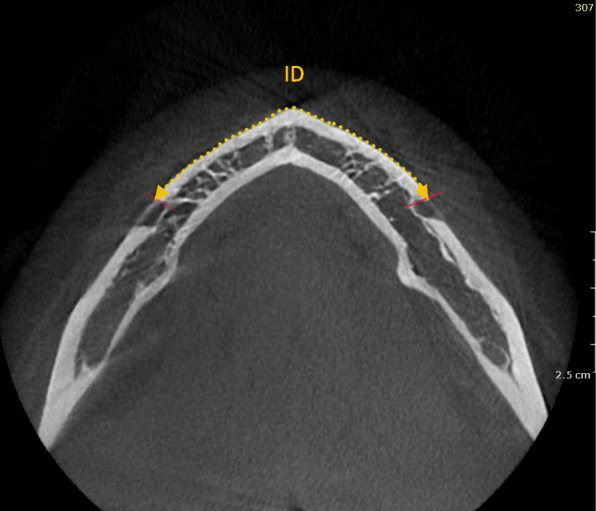
An axial section showing the measurement of interforaminal distance (ID)

The distance between the mesial border of the mental foramen at one side to the mesial border of the mental foramen on the contralateral side at 5 mm caudal to the tooth apex was defined as the ID (Fig. 3). The available distance between the mesial border of the mental foramen and the anterior border of the loop, corresponding to the thinnest part of the mandibular canal, was measured as the anterior loop length in the transverse plane.

The secondary outcome was to evaluate the correlation of the measured parameters with tooth site, age and sex. Therefore, these variables were also recorded.

### Statistical analysis

Data were analyzed by SPSS version 23 (SPSS Inc., IL, USA). Descriptive data were presented as median and interquartile range. The Kolmogorov-Smirnov test was used to assess the normality of data distribution, which showed that the data did not have a normal distribution with 5 % confidence interval. Therefore, in order to evaluate the correlations of the measured parameters with the tooth site, age and sex, the nonparametric tests were applied, namely the Friedman, Mann-Whitney, Wilcoxon, Spearman’s Rho and Kruskal-Wallis tests. P values ≤ 0.05 were considered statistically significant.

## Results

The intraclass correlation coefficient was calculated to be 93 %, indicating excellent intra-examiner reliability.

Of 78 CBCT scans, 39 (50 %) belonged to females and 39 (50 %) belonged to males. The median age of patients was 45 (19.5) years. The median VD, HD, and CT values were 20.21 (3.26), 4.13 (0.37), and 2.25 (0.23) mm, respectively. The median C/C was 1.51 (0.11), the median ID was 52.24 (8.24) mm, and the median anterior loop length was 1.82 (1.06) mm. Details of the data are presented in Table 1.

**Table 1:** Measures of central dispersion for the variables

Parameter	Median	Maximum	Minimum	IQR
VD	20.21	36.10	13.70	3.26
HD	4.13	5.08	2.09	0.37
CT	2.25	3.40	1.19	0.23
C/C	1.51	2.02	1.02	0.11
ID	52.24	58.50	44.10	8.24
LL	1.82	3.40	0.00	1.06

*VD* vertical dimension, *HD* horizontal dimension, *CT* cortical thickness, *C/C* cortical bone to cancellous bone ratio, *ID* interforaminal distance, *LL* anterior loop length, *IQR* interquartile range

The Friedman test revealed significant differences in VD, HD, C/C, and CT among different teeth. Table 2 presents the median values of these parameters according to the type of tooth. HD and CT values had an increasing trend from the central incisors towards premolars such that the highest HD and CT values were measured at the premolar region. The central incisor had the lowest HD and CT values. VD and C/C changes did not follow a regular pattern. Contrary to HD and CT, the maximum and the minimum VD values were related to the central incisor and premolar, respectively. Moreover, the maximum and the minimum C/C values were recorded at the canine and central incisor sites, respectively.

**Table 2:** Measured parameters based on the type of tooth

Parameter	1st Premolar M(IQR)	Canine M(IQR)	Lateral Incisor M(IQR)	Central Incisor M(IQR)	*P*-value
VD	19.86(2.94)	19.49(4.88)	19.96(3.29)	21.08(2.38)	< 0.001
HD	4.50(0.62)	4.30(0.28)	3.97(0.45)	3.70(0.61)	< 0.001
CT	2.64(0.54)	2.20(0.39)	2.14(0.40)	1.76(0.43)	< 0.001
C/C	1.50(0.15)	1.59(0.17)	1.48(0.24)	1.48(0.22)	< 0.001

*VD* vertical dimension, *HD* horizontal dimension, *CT* cortical thickness, *C/C* cortical bone to cancellous bone ratio; *M *median, *IQR* interquartile range

**Fig. 4:**
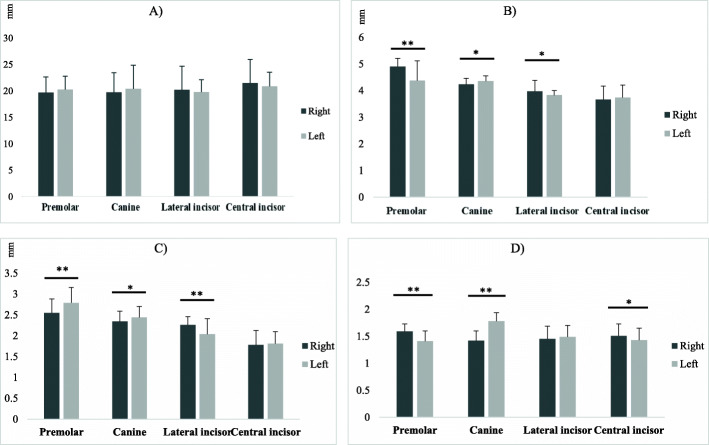
Measured parameters at the site of corresponding teeth with respect to the jaw side. *t significant at *p* < 0.05, **t significant at *p* < 0.01. **A**, Vertical dimension (VD) at the site of corresponding teeth with respect to the jaw side. **B**, Horizontal dimension (HD) at the site of corresponding teeth with respect to the jaw side. **C**, Cortical thickness (CT) at the site of corresponding teeth with respect to the jaw side. **D**, Cancellous to cortical bone ratio (C/C) at the site of corresponding teeth with respect to the jaw side

The Wilcoxon test revealed significant differences in all parameters measured at the canine and premolar regions between the left and right sides of the mandible. Figure 4 shows the measured parameters in the right and left sides of the mandible.

**Fig. 5:**
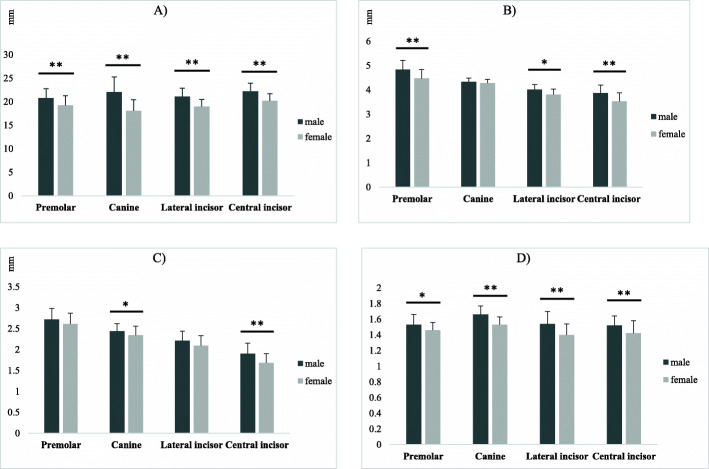
Measured parameters at the site of corresponding teeth with respect to gender. *t significant at *p* < 0.05, **t significant at *p* < 0.01. **A**, Comparison of vertical dimension (VD) between males and females at the site of corresponding teeth. **B**, Comparison of horizontal dimension (HD) between males and females at the site of corresponding teeth. **C**, Cortical thickness (CT) differences between males and females at the site of corresponding teeth. **D**, Cancellous to cortical bone ratio (C/C) differences between males and females at the site of corresponding teeth

All measured parameters were greater in males than females. The Mann-Whitney test showed significant inter-gender differences in all parameters except for HD at the canine region, CT at the premolar and lateral incisor regions, and the anterior loop length, as shown in Fig. 5; Table 3.

**Table 3:** Interforaminal distance and anterior loop length in males and females

Gender	Interforaminal distance M(IQR)	Ant. loop length M(IQR)
Males	55.24(8.86)	2.04(1.01)
Females	49.24(7.80)	1.60(1.12)
*P*-value	0.037*	0.051

*M *median, *IQR* interquartile range; *Statistically significant (*P* value < 0.05)

**Fig. 6:**
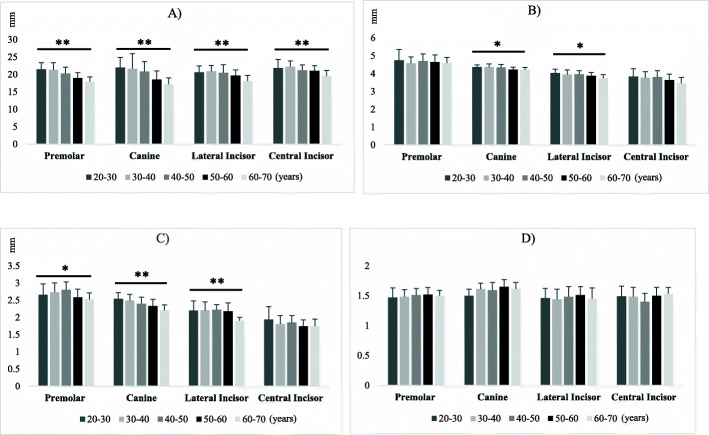
Measured parameters at the site of corresponding teeth in different age groups. *t significant at *p* < 0.05, **t significant at *p* < 0.01. **A**, Vertical dimension (VD) at the site of corresponding teeth in different age groups. **B**, Horizontal dimension (HD) at the site of corresponding teeth in different age groups. **C**, Cortical thickness (CT) at the site of corresponding teeth in different age groups. **D**, Cancellous to cortical bone ratio (C/C) at the site of corresponding teeth in different age groups

According to the Spearman’s Rho, ID significantly increased with age (*p* = 0.000); while, the anterior loop length had a significant inverse correlation with age (*p* = 0.011). The samples were divided into five age groups. The Kruskal-Wallis test revealed significant differences in VD and CT of the corresponding teeth among different age groups. VD had a significant inverse correlation with age (*p* = 0.003). However, it did not follow a regular pattern at the incisor region. No significant difference was found in the C/C ratio of the corresponding teeth according to age group. The difference in HD according to age was only significant for the canine (*p* = 0.008) and lateral incisor teeth (*p* = 0.012). The measured parameters by different age groups are presented in Fig. 6.

## Discussion

The mandibular symphysis is a suitable intraoral donor site for bone graft harvesting due to its easy accessibility and high C/C. Despite the advantages of using the mandibular symphysis for bone harvesting, several complications have also been reported after bone harvesting from the mental region, including intraoperative bleeding, mental nerve injury, and pulp necrosis of the lower anterior teeth. These injuries usually involve anatomical structures such as the mental foramen and apex of the anterior teeth. Therefore, the width, height, and depth of the bone block to be harvested from the symphysis should be precisely determined preoperatively [8].

In this study, HD, VD, CT, and C/C of the mandibular symphysis from the right premolar to the left premolar teeth, ID, and anterior loop length were measured on CBCT scans of 78 patients. Such measurements can be used to define a safe zone for bone harvesting.

The C/C is one determinant of bone quality for bone harvesting. This parameter is highly accurate to determine the revascularization capability. The revascularization rate is faster in the cancellous bone due to the presence of larger bone marrow spaces; while, the high density of the lamellae in the cortical bone decelerates the revascularization process [13]. Despite superior revascularization in the cancellous bone, the cancellous bone resorbs faster under the applied loads [14]. Di Bari et al. evaluated the microstructure of the mandibular symphysis as a donor site for autologous bone harvesting and reported cortical bone volume of 0.71 ± 0.23 mL and cancellous bone volume of 2.16 ± 0.76 mL with a C/C of 3.04 [15]. This ratio was 1.51 ± 0.11 in the present study. The reason for the difference between the ratios may be that Di Bari et al. made measurements based on the volume while our method was based on surface area.

Measurement of the ID is necessary to determine the length of bone block to be harvested. The length of the harvestable bone can be estimated using the ID and anterior loop length, which vary in different patients [16]. Agthong et al. studied 100 mandibles of Thai patients and reported a value of 28 mm for the distance from the mental foramen to midline, corresponding to an ID of 56 mm [17]. Smajilagic et al. evaluated 20 dry mandibles and reported an average ID of 50 mm [18]. The ID was 55.2 mm in a study on Caucasian skulls by Neiva et al. [19]. Lee et al. studied 20 patients with normal occlusion and reported a mean ID of 53.1 ± 3.6 mm [20]. Zeltner et al., [21] and Ataman et al. [22] measured the length of harvestable bone, considering a safe margin of 5 mm away from the mental foramen. Zeltner et al. [21] reported an average ID of 37.46 ± 3.84 mm, and a higher ID value was found in individuals with no missing of molars. Ataman et al. [22] evaluated various intraoral donor sites and concluded that the volume and density of the symphyseal bone were higher than the ramus, palatal and maxillary tuberosity bone blocks. They reported a safe harvestable bone length of 29.76 ± 7.17 mm. The median ID was 52.24 ± 8.24 mm in the present study and was significantly different between males and females, and among different age groups. Agthong et al. found no significant inter-gender difference in ID but this finding was not conclusive due to their small sample size [17]. Lee et al. reported a significant increase in ID with age [20]; while, Kalabalik et al. found no significant difference among different age groups in this respect [23]. The reason for the variations in the results of studies may be the possibility of growth in the third decade of life as well as the differences between the study populations.

The anterior loop of the mandibular canal, which is the anterior extension of the inferior alveolar nerve beyond the mental foramen, is clearly visible on CBCT scans of most patients. Since it contains a collection of nerves and vessels, any injury to the anterior loop in the process of bone harvesting may result in sensory disturbances [24]. Wei et al. found the anterior loop in 67.8 % of the 612 hemimandibles with a mean length of 3.3 ± 1.2 mm [25]. Several studies have underlined the importance of performing osteotomy at a safe distance from the mental foramen [26, 27]. Parnia et al. studied the visibility and length of the anterior loop. The right and left anterior loops were visualized on 83.3 and 62.5 % of the 96 CBCT scans that were re-evaluated, respectively. The mean anterior loop length was 3.54 ± 1.41 mm in their study [28]. This parameter was measured to be 1.82 ± 1.06 mm in the present study; therefore, considering the ID of 52.24 ± 8.24 mm, it is recommended to harvest a bone block with a maximum width of 50.42 mm from this region. The anterior loop is not visualized on CBCT scans of some patients, but the safe distance should not be ignored in such cases.

The HD and CT of the mandibular symphysis increased towards the posterior teeth according to the results of the present study. This increase may allow harvesting larger bone blocks from the posterior regions. The mean HD was 4.5 mm, and the mean CT was 2.3 mm in a study by Lee et al., which increased towards the posterior teeth [20]. The average HD reported by Zeltner et al., [21] and Ataman et al. [22] was 10.27 ± 2.1 mm and 8.38 ± 2.66 mm, respectively. However, the measurement of the horizontal dimension of bone block was different in their studies. They measured the maximum width of the donor site between the buccal surface and 2 mm buccal to the lingual cortex; this explains the higher HD value reported by them. Some studies reported significantly larger values in male patients [29]. Considering the probability of smaller values in female patients, more attention should be paid to these parameters during bone harvesting in such patients. Although deep incisions can be used to determine the CT and minimize the harvesting of cancellous bone, this method is not recommended due to the risk of paresthesia after the procedure [30].

In evaluation of the VD of mandibular symphysis, Montazem et al. recommended a mean height of 9.9 mm for bone harvesting at a safe zone of 5 mm [31]. A safe distance of 5 mm from the inferior border of the symphysis, tooth apices, and anterior border of the mandibular foramen has also been recommended for bone harvesting [16]. Considering the safe distance of 5 mm and the recommendations regarding 9.9 mm height for bone harvesting, a VD of 19.9 mm would be required, which is consistent with a median VD of 20.21 ± 3.26 found in the present study. Zeltner et al. [21] and Ataman et al. [22] considered a safe distance of 5 mm from the tooth apex and 2 mm from the inferior cortex of the mandible. They reported lower VD values (12.71 ± 3.25 mm and 13.36 ± 3.71 mm, respectively). Pommer et al. suggested that the most superior incision for bone harvesting should be at least 8 mm below the tooth apices. This safe distance prevents sensory disturbances in 75 % of patients regardless of the harvesting depth [32]. Lee et al. concluded that patients with larger VD values had larger HD values as well [20]. This correlation was not examined in the present study. However, the results showed a significant difference in VD between male and female patients.

Recommending a safety zone for bone harvesting was not the purpose of this study; however, according to the results of the current study and those of Gandhi V et al., who suggested conservative safe margins of 5mm away from vital structures and inferior cortex of the mandible for bone harvesting [11], bone length and height of 38 and 10 mm, respectively, could be considered for safe bone harvesting. Attention should be paid to the incisive canal in this safe zone to prevent injury to the mandibular incisive nerve and the associated vasculature in the process of bone harvesting. Zeltner et al. [21] reported that the mean distance between the mandibular incisive canal and the apex of the central incisor was 10.5 ± 3.5 mm. Since the use of digital planning is becoming increasingly popular, identifying the planned cuts for bone harvesting would be highly valuable. de Stavola et al. reported 13 cases chosen for autogenous mandibular bone grafting. They used a computer-aided design system to determine the dimensions of bone blocks, which described ideal bone osteotomy planes to facilitate the surgical guide and to reduce the risk of damage to the anatomical structures [33]. Their findings indicated the possibility of performing guided bone block harvesting to obtain an appropriate volume of autogenous bone in a safe manner.

This study had a number of limitations, including a small sample size that affected the significance of the differences in parameters among different age groups and teeth, and also between males and females. Future studies are recommended to measure these parameters in larger populations for more reliable comparisons. Our study presents a different approach for bone quality assessment in CBCT images. This approach can be used in future studies.

## Conclusions

In this study conducted on an Iranian population, the VD, HD and ID, as parameters indicating the bone quantity of the mandibular symphysis, varied among different teeth and between males and females. The parameters representing density or quality of bone namely CT and C/C also varied among different teeth and between males and females. Different age groups also had different VD and CT of mandibular symphysis. Such anatomical differences highlight the importance of a thorough preoperative evaluation to prevent injury to vital anatomical structures and to ensure proper surgical planning. CBCT is an optimal tool to assess the quality and quantity of the available bone for harvesting.

## Data Availability

The datasets used and/or analyzed during the current study are available from the corresponding author on reasonable request.

## References

[1] Kabi S, Kar R, Samal D, Deepak KC, Kar IB, Mishra N (2020). Immediate dental implant placement with or without autogenous bone graft: A comparative study. Natl J Maxillofac Surg.

[2] Sakkas A, Wilde F, Heufelder M, Winter K, Schramm A (2017). Autogenous bone grafts in oral implantology—is it still a “gold standard”? A consecutive review of 279 patients with 456 clinical procedures. Int J Implant Dent.

[3] Giannoudis PV, Dinopoulos H, Tsiridis E (2005). Bone substitutes: an update. Injury.

[4] Zouhary KJ (2010). Bone graft harvesting from distant sites: concepts and techniques. Oral Maxillofac Surg Clin North Am.

[5] Hameed MH, Gul M, Ghafoor R, Khan FR (2019). Vertical Ridge Gain with Various Bone Augmentation Techniques: A Systematic Review and Meta-Analysis. J Prosthodont.

[6] Dolanmaz D, Esen A, Yıldırım G, İnan Ö (2015). The use of autogeneous mandibular bone block grafts for reconstruction of alveolar defects. Ann Maxillofac Surg.

[7] Reininger D, Cobo-Vázquez C, Monteserín-Matesanz M, López-Quiles J (2016). Complications in the use of the mandibular body, ramus and symphysis as donor sites in bone graft surgery. A systematic review. Med Oral Patol Oral Cir Bucal.

[8] Silva FMS, Cortez ALV, Moreira RWF, Mazzonetto R (2006). Complications of intraoral donor site for bone grafting prior to implant placement. Implant Dent.

[9] Tahmaseb A, Wismeijer D, Coucke W, Derksen W (2014). Computer technology applications in surgical implant dentistry: a systematic review. Int J Oral Maxillofac Implants.

[10] Möhlhenrich SC, Heussen N, Ayoub N, Hölzle F, Modabber A (2015). Three-dimensional evaluation of the different donor sites of the mandible for autologous bone grafts. Clin Oral Investig.

[11] Gandhi V, Lowney A, Cardarelli L, Yadav S, Tadinada A (2020). Three-dimensional evaluation of the mandibular symphyseal region in block graft harvesting for dental implants using cone-beam computed tomography. Imaging Sci Dent.

[12] Altug HA, Coskun AT, Kamburoglu K, Zerener T, Gulen O, Sencimen M (2016). Volumetric evaluation of safe zone for bone harvesting from symphysis region by using cone beam computed tomography. Implant Dent.

[13] Iyengar R, Cherukuri N, Patnala C (2018). Reconstruction of Traumatic, Open Supracondylar Femoral Fractures by Autologous Fibular Strut Grafting and Cortico-Cancellous Bone Grafting—A Single-Centre, Observational Study. J Orthop Trauma.

[14] Baldi D, Pesce P, Musante B, Pera F, Fulcheri E, Romano F (2019). Radiological and Histomorphometric Outcomes of Homologous Bone Graft in Postextractive Implant Sites: A 6-Year Retrospective Analysis. Implant Dent.

[15] Di Bari R, Coronelli R, Cicconetti A (2013). Radiographic evaluation of the symphysis menti as a donor site for an autologous bone graft in pre-implant surgery. Imaging Sci Dent.

[16] Al-Ani O, Nambiar P, Ha KO, Ngeow WC (2013). Safe zone for bone harvesting from the interforaminal region of the mandible. Clin Oral Implants Res.

[17] Agthong S, Huanmanop T, Chentanez V (2005). Anatomical variations of the supraorbital, infraorbital, and mental foramina related to gender and side. J Oral Maxillofac Surg.

[18] Smajilagić A, Dilberović F (2004). Clinical and anatomy study of the human mental foramen. Bosn J Basic Med Sci.

[19] Neiva RF, Gapski R, Wang HL (2004). Morphometric analysis of implant-related anatomy in Caucasian skulls. J Periodontol.

[20] Lee KA, Kim MS, Hong JY, Lee JS, Choi SH, Chai JK (2014). Anatomical topography of the mandibular symphysis in the Korean population: a computed tomography analysis. Clin Anat.

[21] Zeltner M, Flückiger LB, Hämmerle CH, Hüsler J, Benic GI (2016). Volumetric analysis of chin and mandibular retromolar region as donor sites for cortico-cancellous bone blocks. Clin Oral Implants Res.

[22] Ataman-Duruel ET, Duruel O, Nares S, Stanford C, Tözüm TF (2020). Quantity and Quality of Intraoral Autogenous Block Graft Donor Sites with Cone Beam Computed Tomography. Int J Oral Maxillofac Implants.

[23] Kalabalik F, Aytuğar E (2019). Localization of the Mandibular Canal in a Turkish Population: a Retrospective Cone-Beam Computed Tomography Study. J Oral Maxillofac Res.

[24] Chen Z, Chen D, Tang L, Wang F (2015). Relationship between the position of the mental foramen and the anterior loop of the inferior alveolar nerve as determined by cone beam computed tomography combined with mimics. J Comput Assist Tomogr.

[25] Wei X, Gu P, Hao Y, Wang J (2020). Detection and characterization of anterior loop, accessory mental foramen, and lateral lingual foramen by using cone beam computed tomography. J Prosthet Dent.

[26] Kuzmanovic DV, Payne AG, Kieser JA, Dias GJ (2003). Anterior loop of the mental nerve: a morphological and radiographic study. Clin Oral Implants Res.

[27] Mardinger O, Chaushu G, Arensburg B, Taicher S, Kaffe I (2000). Anterior loop of the mental canal: an anatomical-radiologic study. Implant Dent.

[28] Parnia F, Moslehifard E, Hafezeqoran A, Mahboub F, Mojaver-Kahnamoui H. Characteristics of anatomical landmarks in the mandibular interforaminal region: a cone-beam computed tomography study. Med Oral Patol Oral Cir Bucal. 2012 May 1;17(3):e420-5. 10.4317/medoral.17520 PMC3476108 22143718

[29] Garvin HM, Ruff CB (2012). Sexual dimorphism in skeletal browridge and chin morphologies determined using a new quantitative method. Am J Phys Anthropol.

[30] Gapski R, Wang HL, Misch CE (2001). Management of incision design in symphysis graft procedures: a review of the literature. J Oral Implantol.

[31] Montazem A, Valauri DV, St-Hilaire H, Buchbinder D (2000). The mandibular symphysis as a donor site in maxillofacial bone grafting: a quantitative anatomic study. J Oral Maxillofac Surg.

[32] Pommer B, Tepper G, Gahleitner A, Zechner W, Watzek G (2008). New safety margins for chin bone harvesting based on the course of the mandibular incisive canal in CT. Clin Oral Implants Res.

[33] De Stavola L, Fincato A, Bressan E, Gobbato L (2017). Results of Computer-Guided Bone Block Harvesting from the Mandible: A Case Series. Int J Periodontics Restorative Dent.

